# Novel finite element‐based plate design for bridging mandibular defects: Reducing mechanical failure

**DOI:** 10.1111/odi.13331

**Published:** 2020-04-14

**Authors:** Bram B. J. Merema, Joep Kraeima, Sebastiaan A. H. J. de Visscher, Baucke van Minnen, Fred K. L. Spijkervet, Kees‐Pieter Schepman, Max J. H. Witjes

**Affiliations:** ^1^ Department of Oral and Maxillofacial Surgery University Medical Center Groningen Groningen The Netherlands

**Keywords:** CAD‐CAM, finite element analysis, mandibular reconstruction, mouth neoplasms, patient‐specific modelling, prosthesis and implants

## Abstract

**Introduction:**

When the application of a free vascularised flap is not possible, a segmental mandibular defect is often reconstructed using a conventional reconstruction plate. Mechanical failure of such reconstructions is mostly caused by plate fracture and screw pull‐out. This study aims to develop a reliable, mechanically superior, yet slender patient‐specific reconstruction plate that reduces failure due to these causes.

**Patients and Methods:**

Eight patients were included in the study. Indications were as follows: fractured reconstruction plate (2), loosened screws (1) and primary reconstruction of a mandibular continuity defect (5). Failed conventional reconstructions were studied using finite element analysis (FEA). A 3D virtual surgical plan (3D‐VSP) with a novel patient‐specific (PS) titanium plate was developed for each patient. Postoperative CBCT scanning was performed to validate reconstruction accuracy.

**Results:**

All PS plates were placed accurately according to the 3D‐VSP. Mean 3D screw entry point deviation was 1.54 mm (*SD*: 0.85, *R*: 0.10–3.19), and mean screw angular deviation was 5.76° (*SD*: 3.27, *R*: 1.26–16.62). FEA indicated decreased stress and screw pull‐out inducing forces. No mechanical failures appeared (mean follow‐up: 16 months, *R*: 7–29).

**Conclusion:**

Reconstructing mandibular continuity defects with bookshelf‐reconstruction plates with FEA underpinning the design seems to reduce the risk of screw pull‐out and plate fractures.

## 1 INTRODUCTION

Patients who require a continuity resection of the mandible due to, for example, head and neck cancer often receive a reconstruction preferably including a free vascularised flap (e.g. fibula graft). However, when a patient's general medical condition does not allow for this type of reconstructive surgery, the mandibular continuity defect can be bridged using solely a conventional reconstruction plate (RP). This type of RP usually needs manual bending to match the contour of the mandible. This method, however, has been reported to fail due to screw loosening or plate fracture (Gellrich et al., 2004; Katakura, Shibahara, Noma, & Yoshinari, 2004; Lopez, Dekeister, Sleiman, & Paoli, 2004; Maurer, Eckert, Kriwalsky, & Schubert, 2010; Schoning & Emshoff, 1998; Shibahara, Noma, Furuya, & Takaki, 2002)**.** Maurer et al. (2010) describe a failure rate of 10% for screw loosening and plate fracture combined, while others report failure solely due to plate fracture in 10% of the cases (Irish et al., 1995; Schoning & Emshoff, 1998; Shibahara et al., 2002) or to screw loosening alone in 18% of the cases (Markwardt, Pfeifer, Eckelt, & Reitemeier, 2007). According to Maurer et al., (2010), all screw loosening occurred within the first 6 months postoperatively.

Plate fracture is seen predominantly in the regions surrounding the screws nearest to the continuity defect and can be caused by fatigue through cyclic in situ loading (Katakura et al., 2004), usually encouraged by residual stress inside the plate as a result of repetitive bending while contouring (Lindqvist et al., 2001; Martola, Lindqvist, Hanninen, & Al‐Sukhun, 2007). It seems to occur mostly in segmental defects that do not cross the midline and in the presence of relatively many remaining occlusal units (Shibahara et al., 2002). Failure of a bridging RP leads to severe discomfort and impaired oral function for the patient (Lindqvist, Soderholm, Laine, & Paatsama, 1992). In most cases, an additional surgical procedure involving a secondary reconstruction is needed (Martola et al., 2007).

Numerous studies applied patient‐specific reconstruction plates (PS‐RP) to prevent plate fracture by changing the material or design of conventional RP (Li et al., 2013; Luo, Xu, Guo, & Rong, 2017; Narra et al., 2014; Singare, Shenggui, & Sheng, 2017) but did not look at screw pull‐out and its prevention. In order to prevent failure in further developed PS‐RP, it is necessary to assess current conventional reconstructions biomechanically. Therefore, this study focused on an analysis of conventional RPs with the finite elements method (FEM), to obtain insight into any weaknesses and to exclude them in further patient‐specific designs.

The aim of this phase 1 study was to design and analyse PS‐RPs to bridge mandibular gaps and to minimise plate fracture and screw pull‐out‐related failure. This was achieved through the development and clinical application of a reconstructive method using a PS‐RP, based on a 3D virtual surgical planning (VSP) and FEM supported individual design.

## 2 PATIENTS AND METHODS

### 2.1 Patients

All the patients included in this study required a reconstruction of either a primary or a secondary mandibular continuity defect due to a fractured or loosened conventional RP. None of these patients' mandible could be reconstructed with a free vascularised bone flap due to the poor quality of the donor site vascularisation, an impaired medical condition or refusal to undergo major free vascularised bone flap surgery. The study was approved by the Medical Ethical board of our centre (no. METc‐2019/301), and informed consent was obtained for all patients.

In the secondary cases, we started by assessing the mechanically failed conventional RPs retrospectively, following plate fracture or screw pull‐out. Postoperative CT scans were used for the segmentation, and 3D models of the mandibular segments were obtained using Mimics 19.0 software (Materialise). In order to perform finite element analysis (FEA) on the failed primary reconstructions, we digitalised the conventional reconstruction plates, which had been bent to follow the mandible's contour. Using the postoperative CT scans of primary reconstructions, we obtained the contours the plates were bent to, and using the manufacturer's dimensions, we designed matching plates for analysis. This was necessary since the quantity of metal artefacts or scatter in the CT data would not allow for proper segmentation of the osteosynthesis material.

**Figure 1:**
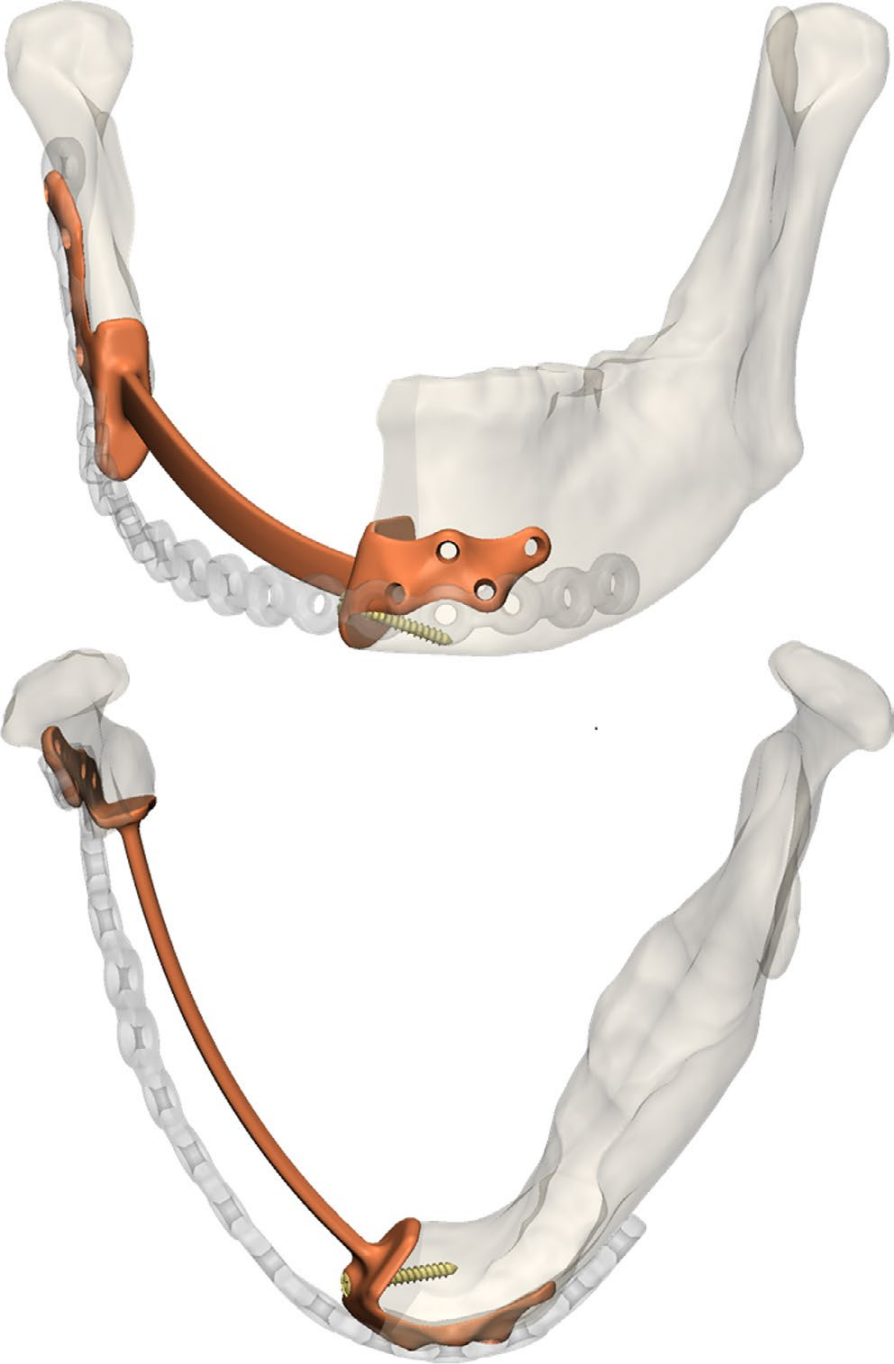
The primary and secondary reconstructions for patient 2. Notice the under‐contouring of the patient‐specific secondary reconstruction plate (orange) compared to the failed primary reconstruction plate (transparent) [Colour figure can be viewed at wileyonlinelibrary.com]

3D FEA (performed with Solidworks Professional 2017 software, Dassault Systèmes Solidworks Corp.) of the conventional reconstructions enabled assessment of the local stress values in the conventional reconstruction plates. The typical defect size used in our general design process was a continuity defect spanning from the mandibular angle up to the approximate midline of the mandible while not crossing it. This represents a L‐defect according to Jewer's HCL classification (Jewer et al., 1989) or a class II defect according to Brown's (Brown, Barry, Ho, & Shaw, 2016) (Figure 1) and is reported to be the most prone to RP failure (Shibahara et al., 2002). In our FEA, we applied the bone material properties and the musculatory system as described by Mesnard and Ramos (Mesnard & Ramos, 2016; Mesnard et al., 2011; Ramos, Ballu, Mesnard, Talaia, & Simões, 2011; Ramos & Mesnard, 2015; Ramos, Nyashin, & Mesnard, 2017). Their models are based on in vivo muscle force measurements and musculature information derived from dissections. They were validated by comparing in silico models to in vitro models of the same human mandibles. In accordance with these studies, incisal bite was simulated since this would ultimately load the mandible. Fixtures were applied to both condyles; thus, the lateral pterygoid muscles were not taken into account. The mandible was assumed to consist of an isotropic cancellous portion, with a cortical outer layer whereby the elastic properties are presented as Young's moduli (Young's modulus measures the stiffness of a solid material) of 400 MPa and 14,700 MPa, respectively (Mesnard & Ramos, 2016). Poisson's ratio, a measure of how a material constricts or expands to a tensile or compressive load, was assumed to be 0.3 for both the cortical and cancellous bone (Mesnard & Ramos, 2016). All the conventional RPs were considered commercially pure grade 2 titanium, with a Young modulus of 102,000 MPa and a Poisson ratio of 0.34, while the PS‐RPs and all the applied screws were assigned 113,800 MPa and 0.34 for Young's modulus and Poisson's ratio, respectively, representing titanium grade 5 alloy (Boyer, Welsch, & Collings, 1994; Holt & Ho, 1996). We used this titanium alloy because of its higher resistance against fatigue, or endurance limit.

### 2.2 Design and production

After analysing the conventional RP, an alternative PS reconstruction method was developed consisting of a 3D virtual surgical plan (VSP) that combines both CT data (i.e. bone segmentation and nerve canal delineation) and fused with MRI‐based tumour delineation for osteotomy placement (Kraeima, Dorgelo, et al., 2018). The aim was to overcome screw pull‐out and high stresses which could lead to plate fracture.

Contrary to the majority of PS‐RP suggested in the literature, which typically consist of a strip‐like plate following the buccal contour of the mandible, we focused on incorporating the osteotomy sites of the mandibular segments for stable fixation of the plate. Bookshelf‐like flanges situated against the osteotomy planes of the mandibular segments were added to a bridging section. To mitigate the chance of dehiscence of the plate due to contraction of the covering soft tissues, the bridging section was under‐contoured with respect to the lateral and caudal boundaries of the preresected mandible (Figure 1). Fixation of the plate was obtained through bi‐cortical screw placement and, whenever possible, mono‐cortically in the flanges supporting the osteotomy sites. The design was carried out in 3‐Matic Medical 11.0 (Materialise). Once finished, the STL file of the plate was exported and converted into a non‐uniform rational basis spline (NURBS) object using Geomagic software (3D Systems). Subsequently, screw threads, compatible with 2.3 locking screws (KLS Martin), were inserted into the NURBS file using the Solidworks Professional 2017 software. Subsequently, the finalised CAD files were sent to the manufacturer (Witec Fijnmechanische Techniek BV) to mill the PS plates from medical grade 5 titanium alloy.

**Figure 2:**
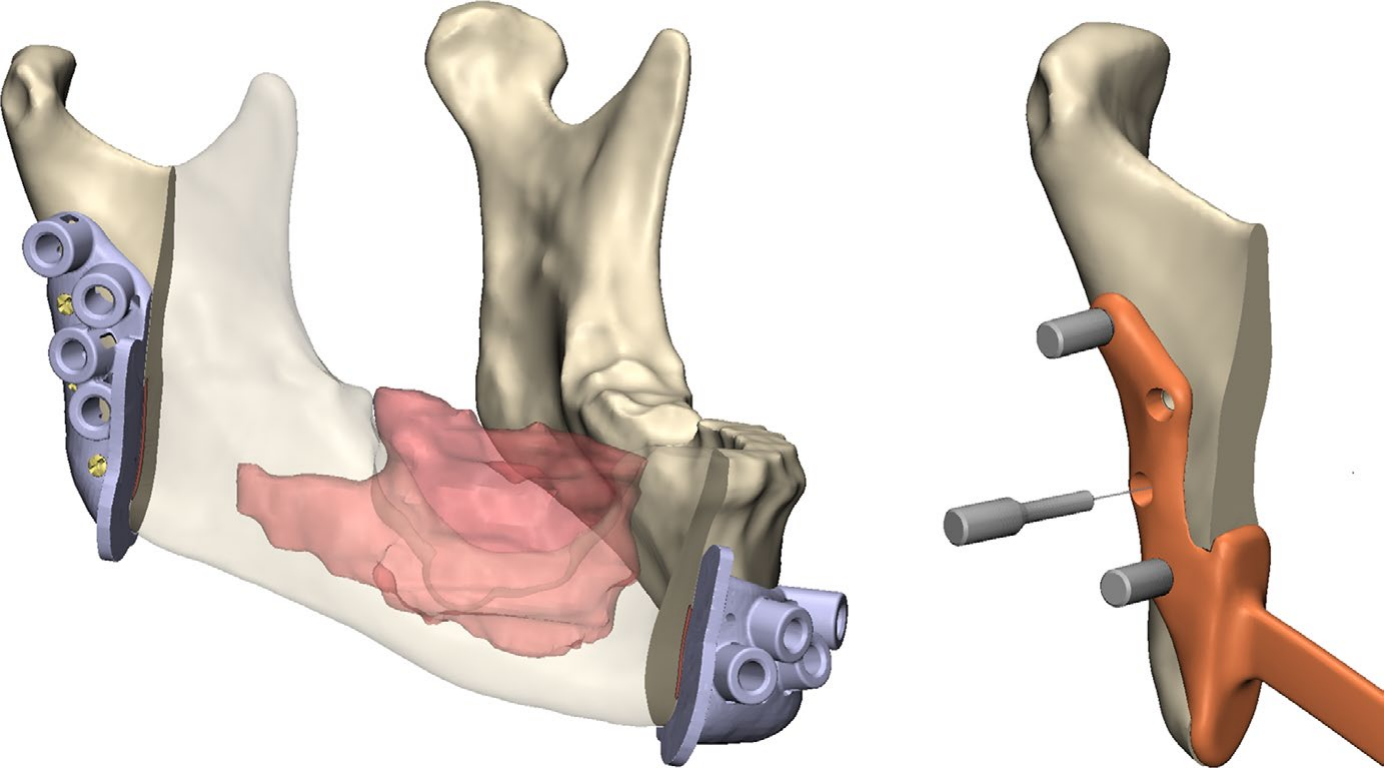
This figure shows an example of the type of surgical guides (top right) and temporary centring pins (lower right) that were used in this study [Colour figure can be viewed at wileyonlinelibrary.com]

In order to accurately translate the VSP to the operating theatre, surgical drill and cutting guides were designed in house and 3D printed by Oceanz (Oceanz BV) from medical grade polyamide powder (Figure 2). Cylinders in the guide indicate the position and direction of the planned screws and function as a pilot drill support when an additional metallic drill sleeve is inserted. Comparable guide designs were applied in previous studies and proved to be an accurate translator of surgical plans (Kraeima, Dorgelo, et al., 2018; Kraeima, Merema, Witjes, & Spijkervet, 2018; Schepers et al., 2015, 2016; Vosselman, Merema, Schepman, & Raghoebar, 2018). In addition, centring pins were designed to function as intraoperative plate positioning devices and inserted into the drilled screws pilot holes (Figure 2).

### 2.3 Measurements

All the patients underwent a postoperative CBCT scan (120 kV/5 mA with a field of view of 130–230 mm and 0.2–0.4 mm voxel size) to assess the accuracy of implant placement by means of screw entry point deviation and angular screw deviation. Manual alignment of the planned 3D objects of the mandibular segments and screw cylinders with the postoperative CBCT was performed with the Mimics Medical 19.0 software. Two observers executed the alignment independently (BJM and JK). The in situ plate was segmented in order to assess angular screw deviation and subsequently matched to the plate's design file, while the manually aligned screw cylinders were moved along. The Geomagic Studio 2012 (3D Systems) software was used for the matching through a best‐fit surface alignment procedure.

Screw entry point deviation was measured between the entry points in the virtual planning, and the cylinders were matched to the postoperative CBCT by means of Euclidean distance (3D) measurements in the 3‐Matic Medical 11.0 software. Prior to these measurements, all the manually aligned mandibular segments and corresponding cylinders were matched to the virtual planning using the global alignment function in 3‐Matic Medical 11.0.

Data analysis was performed using MedCalc for Windows, version 19.0.5 (MedCalc Software). The inter‐observer variability was supported by the calculation of the interclass correlation coefficient (ICC) for every screw entry point, placed by both observers. A value of <.40 is reported as poor, .40–.59 fair, .60–.74 good and .75–1.00 as excellent (Cicchetti, 1994).

## 3 RESULTS

### 3.1 Patients

A total of eight patients who had either already undergone reconstruction of a continuity resection of the mandible or were scheduled to undergo one presented to our centre (*n* = 8). This group required either primary treatment of tumours (*n* = 5) or replacement of a mechanically failed conventional reconstruction plate (*n* = 3). The latter consisted of two patients with a broken conventional 2.7 RP and one patient with pulled out screws, causing loosening of the conventional 2.7 RP and the locking screws. Table 1 shows the detailed overview of these patients. Patients 7 and 8 received postoperative radiotherapy (66 Gy), starting within six weeks after reconstruction with the PS‐RP.

**Table 1:** Patient overview [Colour table can be viewed at wileyonlinelibrary.com]

Patient				Age	Sex	Diagnosis	Indication	PM flap	Follow‐up (months)	Number of screws	Screw entry point dev. (mm)	Screw angular dev. (°)
1		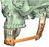	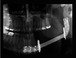	49	♂	Metastasis melanoma mandible	Broken 2.7 OSM plate	No	29	13	2.20 (±0.51)	5.41 (±3.32)
2	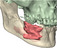	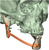	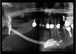	85	♂	T4N0 SCC	Broken 2.7 OSM plate	Yes	25	9	1.49 (±0.42)	4.81 (±2.56)
3	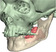	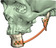	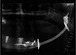	58	♀	T4N0 SCC	Primary recon.	Yes	21	9	1.31 (±0.30)	5.99 (±3.52)
4	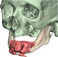	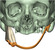	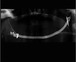	72	♀	T4N0 SCC	Primary recon.	Yes	18	8	0.35 (±0.22)	4.26 (±2.29)
5	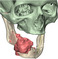	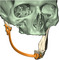	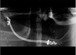	85	♂	T4Nx SCC	Primary recon.	Yes	NA	8	NA	NA
6		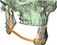	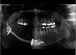	70	♂	Ameloblastoma mandible	Loosened screws 2.7 OSM plates	No	8	8	2.04 (±0.75)	7.49 (±4.27)
7	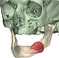	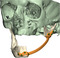	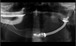	80	♀	T4N0 SCC mandible	Primary recon.	Yes	7	7	0.87 (±0.59)	4.94 (±3.46)
8		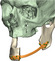	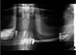	78	♂	Metastasis T2N2 SCC mandible	Primary recon.	Yes	7	10	1.83 (±0.96)	6.29 (±3.20)
Mean				72					16 (*R*: 7–29)	9 (*R*: 7–13)	1.54 (±0.85)	5.76 (±3.27)

Abbreviations: ♀, female; ♂, male; OSM, osteosynthesis material; PM, pectoralis major; SCC, squamous cell carcinoma.

We created comparative FEA of our PS reconstructions for the three patients with failed hand‐bent reconstruction plates. Mandibular segments, loading situations and boundary conditions remained unchanged. The comparative FEA considered the resultant forces on the bone–screw interface, as well as the von Mises or resultant stress occurring in the reconstruction plates. This von Mises stress was used to predict whether or not materials will yield under loading.

### 3.2 Plate fracture

**Figure 3:**
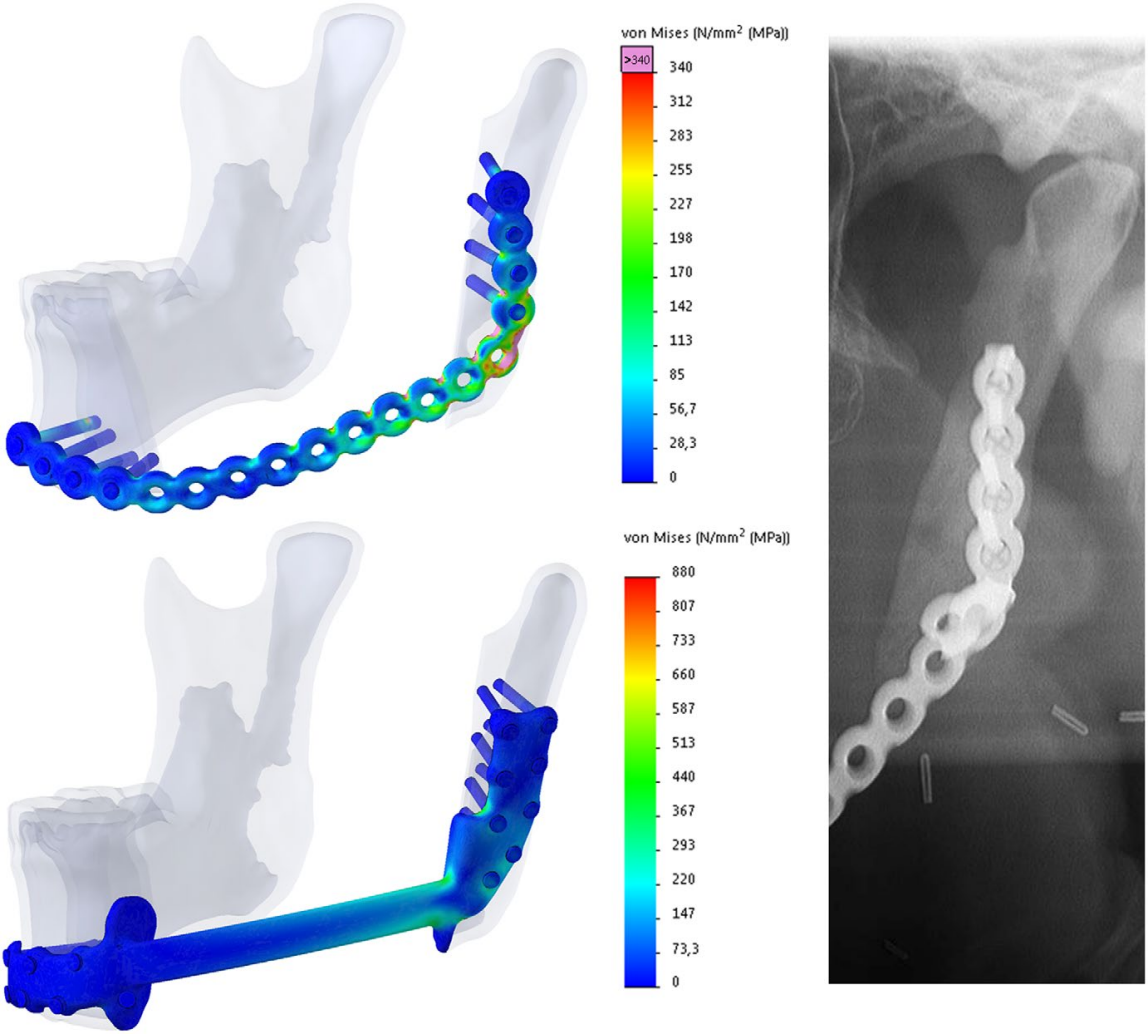
Finite element analysis shows the maximum occurring von Mises stress in both the failed conventional (primary) reconstruction plate (top left) and the PS reconstruction plate (lower left) in patient one and illustrates the resemblance between the highest stress region of the conventional reconstruction plate in silico (note the overloaded pink region in the top left image) and the actual location of the in situ plate fracture (panorex) [Colour figure can be viewed at wileyonlinelibrary.com]

**Figure 4:**
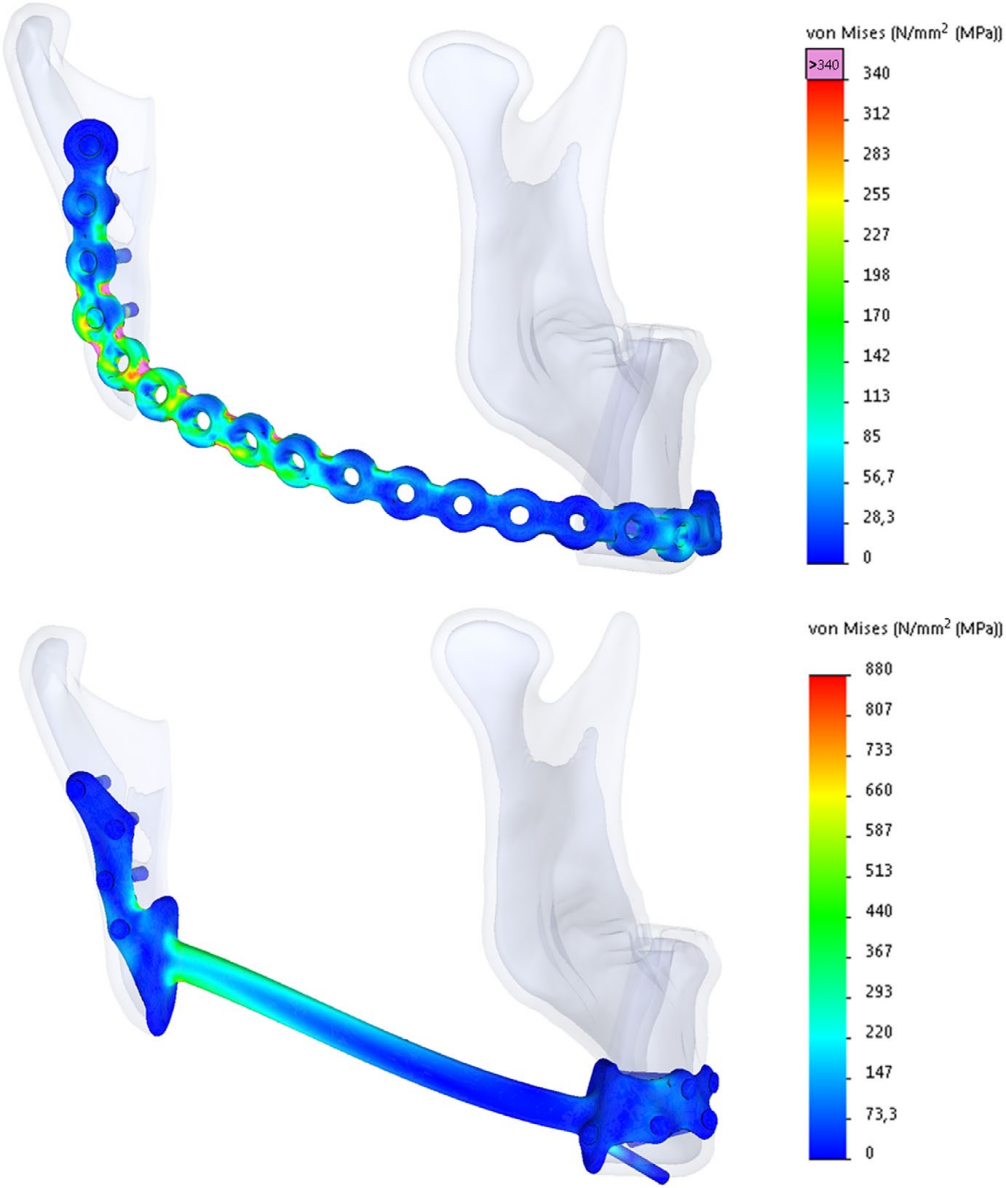
Finite element analysis von Mises stress results for the failed conventional reconstruction (top left) and patient‐specific solution (lower left) for patient 2. The Bridging part height was reduced compared to the first application of our patient‐specific plate, as shown in Figure 2, and remained at this height throughout this study's series [Colour figure can be viewed at wileyonlinelibrary.com]

The FEA results showed that the maximum von Mises stresses in all the analysed conventional RPs exceeded their yield strength (YS), by 42% up to 153%, indicating plastic deformation would occur on applying the load case. The plates would therefore not return to their original shape after loading. Furthermore, the stress in these plates exceeded the material's ultimate strength (US) value by 13% up to 100%, indicating a high risk of plate fracture. The application of our PS reconstruction to the latter patient resulted in a decrease in the YS and US percentages, going from 253% to 59% and 200% to 55%, respectively (Figure 3). In patient number two, who suffered from a fractured conventional RP, we saw a decrease in YS and US percentages, going from 188% to 67% and 149% to 62%, respectively (Figure 4), thereby staying well within acceptable boundaries.

### 3.3 Screw pull‐out

The results of our FEA of the primary conventional reconstruction that failed due to screw pull‐out showed that high resultant forces were acting on these screw surrounding areas. In this case, the RP was fixed at the ventral side of the defect with three screws. Screw one, two and three, counting away from the defect, which had loosened over time, were loaded with 438 N, 416 N and 112 N, respectively. The comparative FEA of our bookshelf‐plate design showed that adding a screw in the ventral bookshelf flange (205 N) would lower the resultant screw forces to 184 N, 144 N and 90 N, respectively, which represents a minimisation of 58%, 66% and 20%. The axial pull‐out force components, the forces along the longitudinal screw directions, did not exceed 180 N for the conventional reconstructions or 90 N for the PS reconstructions.

### 3.4 Surgical procedure

All the PS bookshelf‐reconstruction plates were inserted in accordance with the 3D‐VSP. The surgical procedures were uneventful. During surgery, prior to the drilling of screw pilot holes, the mandibular bone was denuded and the guides were positioned and fixed using 1.5‐mm mini screws (KLS Martin). Subsequently, the osteotomies were performed. Thereafter, the plate was inserted and fitted to the mandibular segments using several centring pins. Once properly aligned, these pins were replaced one by one by 2.3‐mm locking screws with a length in agreement with the surgical planning. Primary closure was performed according to plan in two patients. In the remaining six patients, a pectoralis major flap was used to reconstruct the soft tissue defect and to cover the plate.

### 3.5 Postoperative

Recovery was uneventful from a mechanical point of view for seven patients with a mean of 11.4 days of hospitalisation (*SD*: 9.3, *R*: 3–29). One patient, however, patient 5 in Table 1, deceased in the fourth week postoperatively due to complications related to a PRG probe and therefore could not be followed up. All patients underwent a CBCT scan 6–27 days (mean 12 days) postoperatively. The mean follow‐up period of the 7 patients alive is 16 months (*R*: 7–29) and was uneventful with regard to plate failure or screw pull‐out.

### 3.6 Measurements

Screw entry point deviation (3D) resulted in a mean value of 1.54 mm (*SD* 0.85, *R*: 0.10–3.19) for a total of 64 screws. The mean angular screw deviation was 5.76 degrees (*SD*: 3.27, *R*: 1.26–16.62). The 95% confidence interval of the inter‐observer variability for our measurements was 0.15–0.26 mm with a *P*‐value of .05. The interclass correlation coefficient (two‐way mixed) was 0.97, indicating an excellent match of measurements by both observers.

### 3.7 Dehiscence

**Figure 5:**
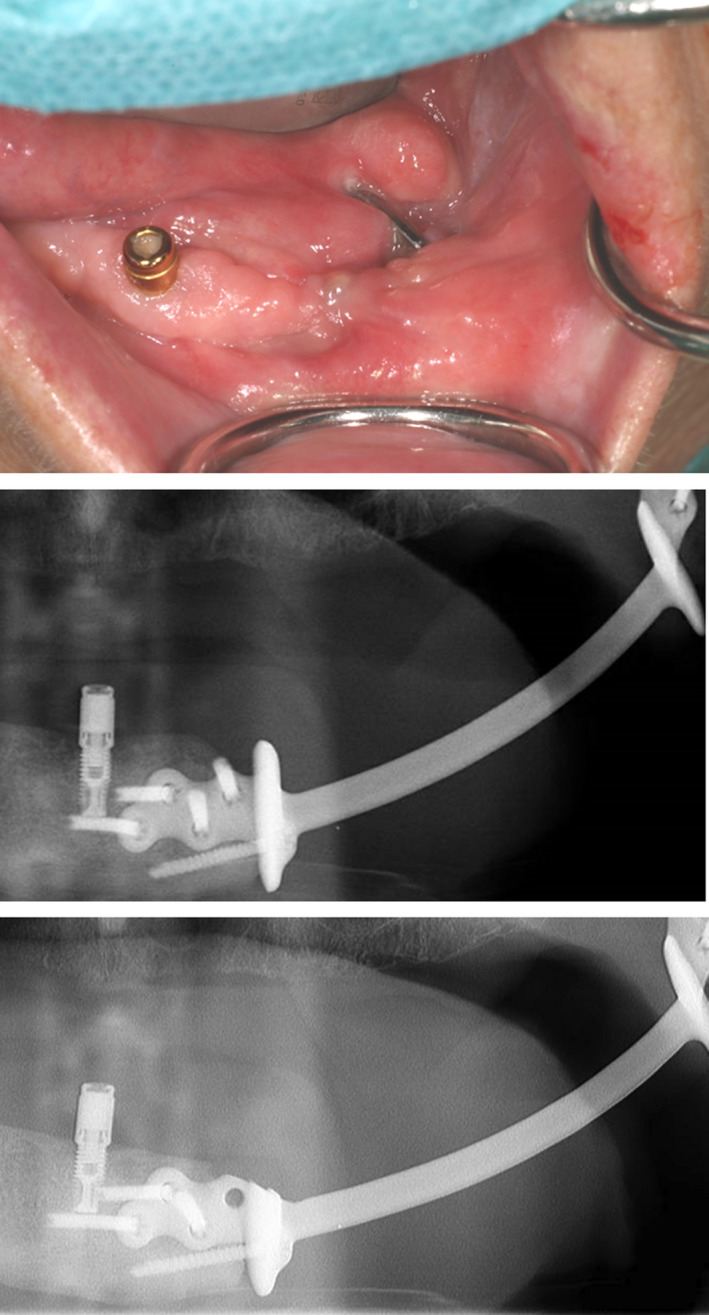
Dehiscence of the cranial section of the ventral bookshelf‐flange in patient 3. The top and middle images are intra‐oral and panorex images, respectively, before surgical bookshelf‐flange modification. The lower panorex image shows the postoperative situation [Colour figure can be viewed at wileyonlinelibrary.com]

The ventral bookshelf‐like flange of our PS‐RPs in the first and third operated patients became partially dehiscent intra‐oral approximately 14 and 4 months postoperatively, respectively. Patient 1 lost 16 kg of bodyweight over a short period of time prior to the intra‐oral dehiscence, which could have played a role in this development. One of these two patients had received a pectoralis major flap, while the other patient underwent primary closure. It was assessed that the design of the flange was too high. The dehiscent cranial part of this flange was surgically removed with some margin (23 and 19 months postoperatively, respectively), and the surrounding soft tissue could be closed (Figure 5). By comparing the 6 days postoperative panorex image of patient 1 to its 18‐month follow‐up, a gradual resorption of the left mandibular angle up to the caudal contour of the plate was observed. There have been no further complications to date.

## 4 DISCUSSION

In this pilot study, we present a unique patient‐specific bookshelf‐reconstruction plate for accurate bridging of mandibular continuity defects. The novel design was based on the FEA of the conventional reconstruction plate failure, with regard to plate fractures and screw loosening. Application of bookshelf‐like flanges, with a screw fixation in the osteotomy sites, resulted in substantial reduction of resultant screw pull‐out inducing forces and, in combination with a change in material, lowered plate stress to within satisfactory levels. Therefore, the chances of the widely reported mechanical problems seen with conventional RPs, which are responsible for 5%–10% of reconstruction failures, were reduced (Gellrich et al., 2004; Katakura et al., 2004; Lopez et al., 2004; Maurer et al., 2010; Schoning & Emshoff, 1998; Shibahara et al., 2002).

Our comparative FEA focused on resultant screw forces rather than the force components in axial screw direction. Most studies found in the literature that look into screw pull‐out describe in vitro results of axial single screw pull‐out. This observation of axial pull‐out could occur with compression screws but not with locking screws, since these have the tendency to be pulled out *en bloc* with the RP rather than axially, due to their semi‐rigid connection with the RP (Cronier et al., 2010). We do know, however, that the axial force components regarding the screws in our study did not exceed 180 N for the conventional reconstructions and 90 N for the PS reconstructions.

The addition of our bookshelf‐flange concept is only of value, in terms of mechanical stability and reduction of the risk of failure, when applied accurately to the planned position. First, a per‐operative visual inspection after guided placement confirmed that no gap remained between the flange and the mandibular bone at the osteotomy sites. Second, postoperative analysis of CBCT scans showed a high accuracy of placement, with 3D deviations comparable to our prior studies and of others, using surgical guides (Kraeima, Dorgelo, et al., 2018; Kraeima, Merema, et al., 2018; Pietruski et al., 2019; Schepers et al., 2016; van Baar, Forouzanfar, Liberton, Winters, & Leusink, 2018; Vosselman et al., 2018). Additionally, careful inspection of the postoperative CBCT scans confirmed contact between the osteotomy sites and bookshelf‐like flanges of the RPs as well as the remainder bone–plate interface, which indicates proper positioning with a small potential shift in the osteotomy plane. The measured screw entry point deviation of 1.58 mm (*SD* 0.82) and angular screw deviation of 5.77 degrees (*SD* 3.33) indicate this method could be applied as a reliable one‐phase procedure for resection and direct reconstruction of a tumour in the mandible. These results represent the accumulation of errors in all visualisation and segmentation steps as well as geometrical errors of the guides and plate and the actual surgical procedure.

Over the last two decades, a rapidly increasing number of PS‐RPs have been presented in the literature with an equal increase in varying finite element analysis models. Applying FEA is of great importance in the design process, since PS‐RPs can still fail mechanically when designed using incorrect assumptions (Li et al., 2014; Luo et al., 2017). Only a very small selection of these FEA models has actually been validated through in vitro and in vivo experiments. Engineers should be always careful when setting up a FEA model, especially complex anatomical models, and all the necessary assumptions that come with it. Also, PS‐RP's should be designed to withstand repetitive loading and the material's fatigue properties for FEA should be chosen accordingly, like we did in this study. Often, only the ultimate or yield properties of a material are taken into account, while the fatigue properties are lower, which could lead to early material failure. We decided to use the most extensively validated model we could find in the literature. However, even this model has its limitations and assumptions. We found that most of the PS‐RPs designs in the literature rely on a strip‐like plate which is positioned and fixated at the buccal contour of the mandible (Gutwald, Jaeger, & Lambers, 2017; Li et al., 2014; Luo et al., 2017; Mazzoni et al., 2013; Narra et al., 2014; Wu, Lin, Liu, & Lin, 2017). We decided to make use of the osteotomy site as well and trap the mandibular segment in between the plate design, which is more stable biomechanically, according to our FEA results.

We expect the dehiscence of the cranial section of the ventral flanges, that occurred in two patients, to be caused by the height of the flanges in combination with contraction of the covering soft tissue. Minimising the bulkiness and height of these flanges might exclude this occurrence. A gradual remodelling of the mandibular angle was observed in one patient past the implant border. The reconstruction plate could have shielded this particular region of the mandible mechanically, causing bone remodelling to occur. Stress‐shielding, also seen in conventional reconstruction plate reconstructions, might explain this resorption. Future application of the topology optimisation technique (Iqbal et al., 2019) could play a key role in minimising the occurrence of, or potentially totally exclude, both stress‐shielding and dehiscence. This engineering technique removes unloaded or unnecessary material and is applied in the FEA phase of the design. It could be used to create geometrically minimalistic designs, while approaching displacement or stress limits, and could prevent a RP from being too stiff, which can cause stress‐shielding, and plates becoming bulky. This study describes a first step in the optimisation of patient‐specific plate design. By allowing for freeform organic structures through topology optimisation, we expect we can lift our patient‐specific reconstructions to a higher level of patient specificity in the near future. Also, based on the results of this phase‐one study we aim to start a multicentre phase‐two study in which we can further validate the effect of our reconstruction method in more patients and over a longer follow‐up period.

## 5 CONCLUSION

Using the finite element method, we retrospectively analysed mechanically failed conventional reconstruction plates and developed an alternative reconstruction plate for the mandible that reduces the chance of screw pull‐out and plate fracture. During this phase‐one study, we successfully reconstructed mandibular continuity defects using our bookshelf‐reconstruction plate concept in eight patients and no mechanical failures have occurred in the study cohort. This novel design of reconstructive plates seems to reduce the risk of screw pull‐out and plate fractures.

## CONFLICT OF INTEREST

There is no conflict of interest to report.

## AUTHOR CONTRIBUTIONS

All authors were involved in the study design and critical reading of the manuscript. Merema and Kraeima analysed the data and Merema, Kraeima and Witjes dr.

## Supporting information

Video S1
